# Large-scale Purification of Type III Toxin-antitoxin Ribonucleoprotein Complex and its Components from *Escherichia coli* for Biophysical Studies

**DOI:** 10.21769/BioProtoc.4763

**Published:** 2023-07-05

**Authors:** Parthasarathy Manikandan, Kavyashree Nadig, Mahavir Singh

**Affiliations:** Molecular Biophysics Unit, Indian Institute of Science, Bengaluru, 560012, India

**Keywords:** Toxin–antitoxin system, ToxIN, RNA–protein complex, Type III TA system, Co-purification

## Abstract

Toxin–antitoxin (TA) systems are widespread bacterial immune systems that confer protection against various environmental stresses. TA systems have been classified into eight types (I–VIII) based on the nature and mechanism of action of the antitoxin. Type III TA systems consist of a noncoding RNA antitoxin and a protein toxin, forming a ribonucleoprotein (RNP) TA complex that plays crucial roles in phage defence in bacteria. Type III TA systems are present in the human gut microbiome and several pathogenic bacteria and, therefore, could be exploited for a novel antibacterial strategy. Due to the inherent toxicity of the toxin for E. coli, it is challenging to overexpress and purify free toxins from E. coli expression systems. Therefore, protein toxin is typically co-expressed and co-purified with antitoxin RNA as an RNP complex from E. coli for structural and biophysical studies. Here, we have optimized the co-expression and purification method for ToxIN type III TA complexes from E. coli that results in the purification of TA RNP complex and, often, free antitoxin RNA and free active toxin in quantities required for the biophysical and structural studies. This protocol can also be adapted to purify isotopically labelled (e.g., uniformly 15N- or 13C-labelled) free toxin proteins, free antitoxin RNAs, and TA RNPs, which can be studied using multidimensional nuclear magnetic resonance (NMR) spectroscopy methods.

Key features

Detailed protocol for the large-scale purification of ToxIN type III toxin–antitoxin complexes from *E. coli*.

The optimized protocol results in obtaining milligrams of TA RNP complex, free toxin, and free antitoxin RNA.

Commercially available plasmid vectors and chemicals are used to complete the protocol in five days after obtaining the required DNA clones.

The purified TA complex, toxin protein, and antitoxin RNA are used for biophysical experiments such as NMR, ITC, and X-ray crystallography.

**Graphical overview**


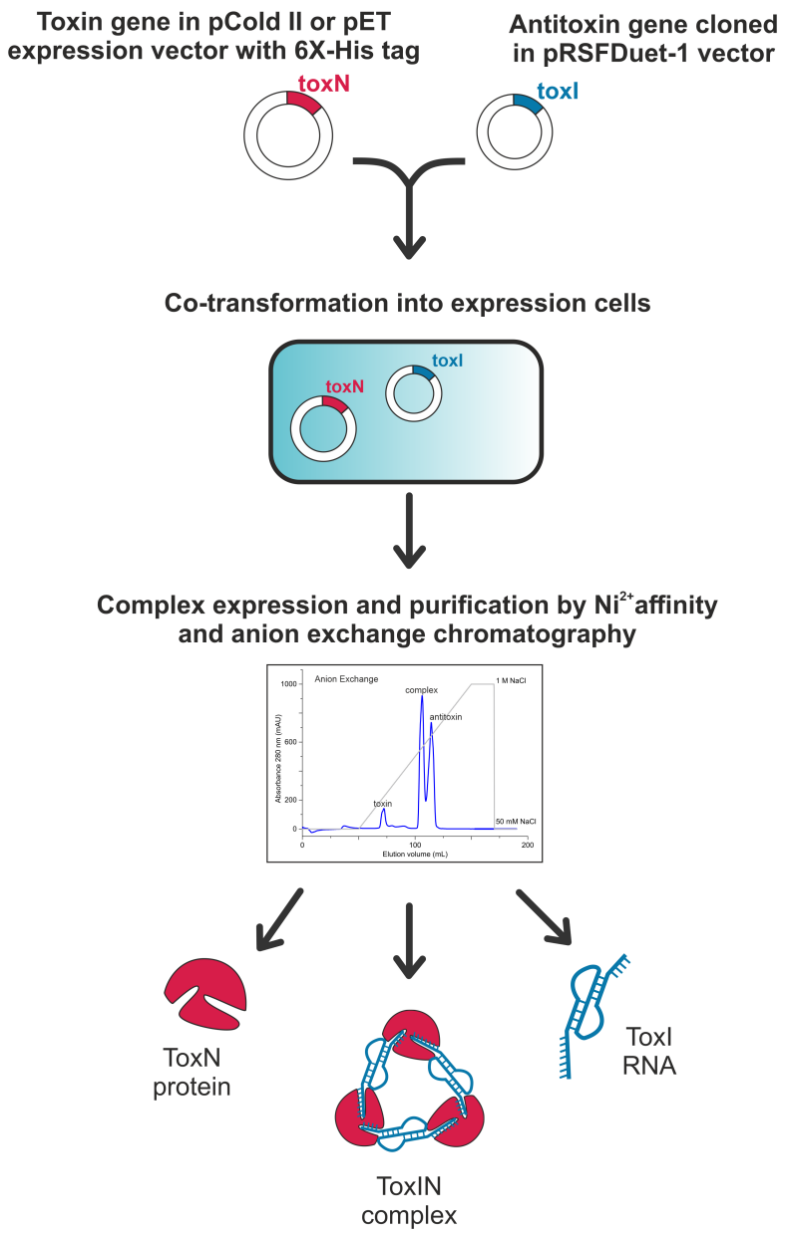


## Background

Type III toxin–antitoxin (TA) systems are genetic modules found in several bacteria, consisting of a protein toxin and a noncoding RNA antitoxin (Fineran et al., 2009). Antitoxin RNA repeats directly bind to the protein toxin, inhibiting its activity by forming a TA ribonucleoprotein (RNP) complex under homeostatic conditions. The primary functions of type III TA systems are attributed to the phage inhibition mechanism in bacteria (Song and Wood, 2020; LeRoux and Laub, 2022; Lin et al., 2023). Type III TA systems are widespread and present in several pathogenic bacteria, including *Staphylococcus aureus, Yersinia pseudotuberculosis*, and *Fusobacterium nucleatum* (Blower et al., 2012), and several bacteria in the gut microbiome. Thus, understanding the structure and assembly of type III TA systems could help in devising novel antibacterial strategies by targeting these systems to free the toxins in the pathogenic bacteria, resulting in bacterial growth arrest or death.

The type III TA systems are classified into three families: ToxIN, CptIN, and TenpIN, based on toxin sequence homology (Blower et al., 2012). Among these classes, the ToxIN family (here, protein toxin and antitoxin RNA are called ToxN and ToxI, respectively) is the most studied in terms of its structure, assembly, and functions (Blower et al., 2011; Short et al., 2012; Guegler and Laub, 2021; Manikandan et al., 2022). In a recent study, we classified *E. coli* ToxIN TA systems into five clusters based on the sequence analysis of the toxin proteins (Manikandan et al., 2022). The structural and functional analysis of a CptIN TA complex from *Eubacterium rectale* has also been reported (Rao et al., 2015).

It is often difficult to purify free toxins from *E. coli* due to their inherent toxicity to the bacteria. To circumvent this issue, toxin and antitoxin are often co-expressed and co-purified as non-toxic TA complexes. This is followed by denaturing the TA complex (e.g., in the presence of 6 M guanidinium hydrochloride or 8 M urea) to separate the toxin and antitoxin. The free denatured toxin can then be refolded back to its natural form. However, in vitro refolding of proteins is not always successful. Alternatively, in some cases, active-site toxin mutants have been generated, which can be expressed in *E. coli* and purified (Samson et al., 2013). While several experiments can be performed using the catalytic mutant protein, a wild-type active protein is often still required to understand the toxins’ mechanism of action.

Here, by adapting the methods published earlier (Blower et al., 2011; Short et al., 2012; Rao et al., 2015; Manikandan et al., 2022), we have optimized a protocol to co-express and co-purify the ToxIN complexes and their components from three clusters of ToxIN family from *E. coli* (Manikandan et al., 2022) for their biophysical and structural investigation [using X-ray crystallography and nuclear magnetic resonance (NMR) spectroscopy]. The method described here yields TA RNP complex, free protein toxin, and free antitoxin RNA of the ToxIN system with an approximate molar ratio of 3:2:2 (with an approximate yield of 7, 3, and 1.5 mg, respectively, from one litre of LB media). The availability of a robust purification method could potentially break the barrier between the discovery of new type III TA systems and their subsequent biophysical characterization to understand their mechanism of action.

## Materials and reagents

**Biological materials**

1. *E. coli* BL21(DE3) competent cells (prepared in the lab following a standard protocol) (Sambrook and Russell, 2006)
2. *E. coli* DH5α competent cells (prepared in the lab following a standard protocol)
3. pCold^TM^ II vector (Takara Bio, catalog number: 3362)
4. pET-21a(+) (Novagen, catalog number: 69740-3)
5. pETDuet^TM^-1 vector (Novagen, catalog number: 71146-3)
6. pRSFDuet^TM^-1 vector (Novagen, catalog number: 71341-3)

**Reagents, chemicals, and kits**

1. Acetic acid (glacial) (Sisco Research Laboratories, catalog number: 85801, CAS number: 64-19-7)
2. Acrylamide:Bis-acrylamide (19:1) for electrophoresis, 40% solution (Fischer BioReagents^TM^, catalog number: BP1406-1, CAS number: 79-06-1), store at 4–8 °C
3. Agarose low EEO (Sisco Research Laboratories, catalog number: 36601, CAS number: 9012-36-6)
4. Ammonium persulfate (APS) (Sisco Research Laboratories, catalog number: 65553, CAS number: 7727-54-0)
5. Ampicillin (Sisco Research Laboratories, catalog number: 61314, CAS number: 69-52-3), store at 2–8 °C
6. Bradford reagent (Sigma, catalog number: B6916-500 mL), store at 2–8 °C
7. Bromophenol Blue indicator (Sisco Research Laboratories, catalog number: 11458, CAS number: 115-39-9)
8. Coomassie brilliant blue (Sisco Research Laboratories, catalog number: 93473, CAS number: 6104-58-1)
9. D/L-Dithiothreitol (DTT) (Sisco Research Laboratories, catalog number: 17315, CAS number: 3483-12-3), store at 0–4 °C
10. dNTPs (New England Biolabs, catalog number: N0447S), store at -20 °C
11. DpnI (New England Biolabs, catalog number: R0176), store at -20 °C
12. Ethanol (Changshu Hongsheng Fine Chemicals, analytical grade, UN No: 1170)
13. Ethylenediaminetetraacetic acid (EDTA) (Sisco Research Laboratories, catalog number: 50027, CAS number: 6381-92-6)
14. Formamide (Sisco Research Laboratories, catalog number: 71714 (062930), CAS number: 75-12-7)
15. Gel extraction kit (QIAquick^®^, Qiagen, catalog number: 28704)
16. Glycerol (Sisco Research Laboratories, catalog number: 62417, CAS number: 56-81-5)
17. Glycine (Sisco Research Laboratories, catalog number: 64072, CAS number: 56-40-6)
18. Hydrochloric acid (Sisco Research Laboratories, catalog number: 65955, CAS number: 7647-01-0)
19. Imidazole (Sisco Research Laboratories, catalog number: 61510-500G)
20. Isopropyl β-d-1-thiogalactopyranoside (IPTG) (Sisco Research Laboratories, catalog number: 67208, CAS number: 367-93-1), store at 0–4 °C
21. Kanamycin (Sisco Research Laboratories, catalog number: 99311, CAS number: 25389-94-0), store at 2–8 °C
22. Luria Bertani agar (LA), Miller (Himedia, catalog number: M1151-500G)
23. Luria broth (LB) (Himedia, catalog number: M575-500G)
24. Methanol for HPLC (SD Fine Chemical Limited, catalog number: 25217 L25)
25. NaCl (Sisco Research Laboratories, catalog number: 41721, CAS number: 7647-14-5)
26. NcoI (New England Biolabs, catalog number: R3193S), store at -20 °C
27. NdeI (New England Biolabs, catalog number: R0111S), store at -20 °C
28. PCR purification kit (Qiagen, QIAquick^®^ PCR purification kit, catalog number: 28104)
29. Phusion High-Fidelity DNA polymerase (New England Biolabs, catalog number: M0530S), store at -20 °C
30. Plasmid extraction kit (QIAprep^®^ spin Miniprep kit, Qiagen, catalog number: 27106)
31. Protease inhibitor cocktail (Roche, catalog number: 04693159001), store at 2–8 °C
32. Quick calf intestinal phosphatase (CIP) (New England Biolabs, catalog number: M0525S), store at -20 °C
33. Sodium dodecyl sulphate (SDS) (Sisco Research Laboratories, catalog number: 32096, CAS number: 151-21-3)
34. T4 DNA ligase (New England Biolabs, catalog number: M0202S), store at -20 °C
35. Tetramethylethylenediamine (TEMED) (Spectrochem private limited, catalog number: 012017), store at 2–8 °C
36. Toluidine Blue (Sisco Research Laboratories, catalog number: 22134, CAS number: 92-32-9)
37. Tris (Sisco Research Laboratories, catalog number: 71033-500G)
38. Urea (Sisco Research Laboratories, catalog number: 21113, CAS number: 57-13-6)
39. XhoI (New England Biolabs, catalog number: R0145S), store at -20 °C
40. β-Mercaptoethanol (Sisco Research Laboratories, catalog number: 83759, CAS number: 60-24-2)

**Plastic and other materials**

1. Centrifuge bottle 500 mL (Tarsons, catalog number: 544020)
2. Centrifuge tubes 15 mL (Tarsons, Spinwin^TM^ Tube Conical bottom, catalog number: 520060)
3. Centrifuge tubes 50 mL (Tarsons, catalog number: 520061)
4. Membrane filters (0.22 μm) (Merck Life Science private limited, catalog number: GVWP04700)
5. Membrane filters (0.45 μm) (Merck Life Science private limited, catalog number: HVLP04700)
6. Microcentrifuge tubes 1.5 mL (Tarsons, catalog number: 500010)
7. Microcentrifuge tubes 2 mL (Tarsons, catalog number: 500020)
8. Oakridge centrifuge tubes (50 mL) (Thermo Scientific, catalog number: 3115-0050)
9. PCR tubes 0.2 mL flat cap (Tarsons, catalog number: 510051)
10. SnakeSkin dialysis bag 3.5 kDa cut off (dialysis bag) (Thermo Scientific, catalog number: 88242), store at 2–8 °C
11. Syringe filters (0.22 μm) (Sartorius Minisart^®^, catalog number: S6534-FMOSK)
12. Syringe filters (0.45 μm) (Sartorius Minisart^®^, catalog number: S6555-FMOSK)
13. Syringes 10 mL (Hindustan Syringes and Medical Devices Limited, Dispo Van, India)
14. Syringes 20 mL (Hindustan Syringes and Medical Devices Limited, Dispo Van, India)

**solutions**

1. PCR reaction mixture for all the amplifications (see Recipes)
2. Double digestion of vector and insert using restriction enzymes (see Recipes)
3. Ligation reaction of vector and insert (see Recipes)
4. Lysis buffer (see Recipes)
5. Wash buffer (see Recipes)
6. Elution buffer (see Recipes)
7. Final wash buffer (see Recipes)
8. Dialysis buffer (see Recipes)
9. Ion exchange chromatography buffers (see Recipes)
10. Size exclusion chromatography buffer (see Recipes)
11. Separating gel for 12% SDS-PAGE (see Recipes)
12. Stacking gel for SDS-PAGE (see Recipes)
13. 5× loading dye for SDS-PAGE (see Recipes)
14. Staining solution for SDS-PAGE (see Recipes)
15. 10× running buffer for SDS-PAGE (see Recipes)
16. SDS-PAGE destaining solution (see Recipes)
17. Urea-acrylamide gel (see Recipes)
18. Formamide dye (see Recipes)
19. SDS-loading dye (5×) (see Recipes)
20. Tris-borate-EDTA (TBE) buffer (see Recipes)

## Recipes

1. **PCR reaction mixture for all the amplifications**
  **Table:** 

Reaction component	Volume (μL) (per 20 μL reaction)
5× Phusion HF buffer	4
10 mM dNTPs	0.4
10 μM forward primer	1
10 μM reverse primer	1
Template DNA (100 ng/μL)	1
Autoclaved MilliQ H_2_O	12.4
Phusion^®^ High-Fidelity DNA Polymerase	0.2
Total volume	20
2. **Double digestion of vector and insert using restriction enzymes**
  **Table:** 

Reaction component	Volume (μL) (per 50 μL reaction)
10× Cutsmart buffer	5
Enzyme 1	1
Enzyme 2	1
Vector/insert DNA	5 μg/2 μg
Autoclaved MilliQ H_2_O	make up to 50 μL
Total volume	50
3. **Ligation reaction of vector and insert**
  **Table:** 

Reaction component	Volume (μL) (per 20 μL reaction)
10× ligase buffer	2
Digested purified vector (10 ng/μL) Insert (10 ng/μL)	5 5
T4 DNA ligase Autoclaved MilliQ H_2_O	1 7
Total volume	20
4. **Lysis buffer**
  **Table:** 

Reagent	Final concentration	Amount
NaCl (4 M)	300 mM	37.5 mL
Tris-HCl (1 M, pH 7.5) Imidazole (5 M, pH 8.0) Glycerol (100%) β-mercaptoethanol (14.28 M)	50 mM 10 mM 10% 2 mM	25 mL 1 mL 50 mL 70 μL
MilliQ H_2_O	n/a	386.43 mL
Total	n/a	500 mL
5. **Wash buffer**
  **Table:** 

Reagent	Final concentration	Amount
Lysis buffer	n/a	49.9 mL
Imidazole (5 M, pH 8.0)	20 mM	100 μL
Total	n/a	50 mL
6. **Elution buffer**
  **Table:** 

Reagent	Final concentration	Amount
Lysis buffer	n/a	24 mL
Imidazole (5 M, pH 8.0)	200 mM	1 mL
Total	n/a	25 mL
7. **Final wash buffer**
  **Table:** 

Reagent	Final concentration	Amount
Lysis buffer	n/a	18 mL
Imidazole (5 M, pH 8.0)	500 mM	2 mL
Total	n/a	20 mL
8. **Dialysis buffer**
  **Table:** 

Reagent	Final concentration		Amount
NaCl (4 M)	50 mM		25 mL
Tris-HCl (1 M, pH 7.5)	50 mM	100 mL	
DTT	1 mM	308.48 mg	
MilliQ H_2_O	n/a		1,875 mL
Total	n/a		2 L
9. **Ion-exchange chromatography buffers**
  Buffer A: same as dialysis buffer.
  Buffer B:
  **Table:** 

Reagent	Final concentration		Amount
NaCl (4 M)	1 M		125 mL
Tris-HCl (1 M, pH 7.5)		50 mM	25 mL
DTT		1 mM	77.12 mg
MilliQ H_2_O	n/a		350 mL
Total	n/a		500 mL
10. **Size-exclusion chromatography buffer**
  **Table:** 

Reagent	Final concentration			Amount
NaCl (4 M)	50 mM			6.25 mL
Tris-HCl (1 M, pH 7.5)		20 mM	10 mL	
DTT		1 mM	77.12 mg	
MilliQ H_2_O	n/a			483.75 mL
Total	n/a			500 mL
11. **Separating gel for 12% SDS-PAGE (prepare solution volume depending on the gel cast size)**
  **Table:** 

Reagent	Amount
Acrylamide:Bis-acrylamide (19:1) (40% solution)	2.4 mL
1.5 M Tris, pH = 8.8 10% SDS solution 10% APS solution TEMED MilliQ H_2_O	2 mL 80 μL 80 μL 8 μL 3.432 mL
Total	8 mL
12. **Stacking gel for SDS-PAGE (prepare solution volume depending on the gel cast size)**
  **Table:** 

Reagent	Amount
Acrylamide:Bis-acrylamide (19:1) (40% solution)	0.75 mL
1.5 M Tris, pH = 6.8 10% SDS solution 10% APS solution TEMED MilliQ H_2_O	1.25 mL 50 μL 50 μL 5 μL 2.9 mL
Total	5 mL
13. **5× loading dye for SDS-PAGE**
  **Table:** 

Reagent	Amount
β-Mercaptoethanol Bromophenol Blue Glycerol SDS Tris-Cl (pH = 6.8)	5% 0.02% 30% 10% 250 mM
14. **Staining solution for SDS-PAGE**
  **Table:** 

Reagent	Amount
Coomassie brilliant blue	1 g
Methanol Acetic acid MilliQ H_2_O	400 mL 100 mL 500 mL
Total	1 L
15. **10× running buffer for SDS-PAGE**
  **Table:** 

Reagent	Amount
Tris base	30 g
Glycine SDS MilliQ H_2_O	144 g 10 g Make up to 1L
Total	1 L
16. **SDS-PAGE destaining solution**
  **Table:** 

Reagent	Amount
Methanol	40 mL
Acetic acid (glacial) MilliQ H_2_O	10 mL 50 mL
Total	100 mL
17. **Urea-acrylamide gel (prepare solution volume depending on the gel cast size)**
  **Table:** 

Reagent	Concentration
Acrylamide:Bis-acrylamide (19:1)	15%
Urea	8 M
TBE buffer	1×
APS	1%
TEMED	0.1%
MilliQ H_2_O	Make up the volume as per requirement
18. **Formamide dye**
  Prepared using a CSHL protocol for formamide gel-loading buffer (Cold Spring Harb Protoc, 2013)
19. **SDS-loading dye (5×)**
  Prepared using a CSHL protocol (Cold Spring Harb Protoc, 2008)
20. **Tris-borate-EDTA (TBE) buffer**
  Prepared according to a CSHL protocol (Cold Spring Harb Protoc, 2010)

## Equipment

1. Amicon Ultra-15 centrifugal filter unit, 3.5 kDa cutoff (Millipore Sigma, catalog number: UFC9030)
2. Anion-exchange column (HiTrap^TM^, Q FF, 5 mL)
3. Centrifuge (Kubota, Model-6500, serial number: K60115-G000)
4. Centrifuge for 50 and 1 mL tubes (Eppendorf, model: 5804R)
5. Dry bath (Bionova, model: SLM-DB-120)
6. Electrophoresis equipment (BIOBEE^®^ Tech)
7. Electrophoresis power supply (Bio-Rad, PowerPac^TM^ Basic, 041BR178399)
8. Fast protein liquid chromatography (FPLC), with fraction collector (GE Healthcare, ÄKTA prime plus)
9. Laminar airflow (local make)
10. Mastercycler (Eppendorf Flexi lid Nexus, 6333, serial number: 6333DQ408627)
11. Mixer (Eppendorf Mixmate 22331 Hamburg, serial number: 5353DN316975)
12. NanoDrop (Thermo Scientific, NanoDrop 2000c spectrophotometer)
13. Ni^2+^-NTA column (GE Healthcare HisTrap^TM^ HP, 5 mL)
14. Peristaltic pump (Bio-Rad, Econo gradient pump, serial number: 491BR 1897, catalog number: 731-9002)
15. pH meter (Thermo Scientific^TM^ Orion Star^TM^ A111)
16. Rotor (Kubota, 6 × 50 mL- AG-506R)
17. Rotor (Kubota, 6 × 500 mL- AG-5006A)
18. S200 column (HiLoad^TM^ 16/600 superdex^TM^ 200pg, ID-0059)
19. SDS-PAGE setup (Invitrogen, Minigel tank)
20. Shaker and incubator (BioTek, NB-205VQ)
21. Sonicator (LABMAN Scientific Instruments, model: pro650)
22. Spectrophotometer (Eppendorf Biophotometer D30, serial number: 6133D0400689)
23. Tabletop centrifuge (Eppendorf centrifuge 5418, FA-45-18-11 S/N:20881)
24. Vacuum pump (Tarsons, Rockyvac 400)
25. Water filtration source (Millipore, Sigma)
26. Weighing balance, milligram sensitivity (Sartorius, BSA 623S-CW)

## Procedure

- **Molecular cloning of type III antitoxin RNA**
  1. Perform the DNA synthesis and cloning of the identified type III TA operon into a high copy number *E. coli* plasmid vector through a gene synthesis service provider. Sub-clone the antitoxin repeats and its natural promoter into pRSFDuet-1 vector using standard cloning protocols described below (Figure 1A and Supplementary Table 1).
  2. Design forward and reverse primers to amplify the antitoxin region along with the natural promoter and terminator, with addition of restriction enzyme sites NcoI and XhoI at the 5′ and 3′ ends of the insert. PCR amplify the insert from the plasmid containing type III TA operon, using Phusion DNA polymerase (a PCR reaction setup is indicated in Recipe 1 and Table 1).
    **Table 1.** Thermocycling conditions for the PCR reaction

Step	Temperature (°C)	Duration	No. of cycles
Initial denaturation	98	30 s	1
Denaturation	98	10 s	30
Annealing	T_a_	30 s	
Extension	72	30 s per kb	
Final extension	72	10 min	1
Hold	4	∞	-
  3. Purify the PCR-amplified insert using the PCR purification kit following the manufacturer’s protocol and elute DNA using nuclease-free water.
  4. Digest ~1–2 μg of the PCR purified insert using the restriction enzymes NcoI and XhoI by incubating at 37 °C for 2 h by setting up a reaction as described in Recipe 2.
  5. Digest ~5 μg of the pRSFDuet-1 vector using the same restriction enzymes in a 50 μL reaction. After digesting the vector, incubate the reaction mixture at 80 °C for 20 min to heat-inactivate the restriction enzymes. Treat the mixture with 0.3 μL of calf intestinal phosphatase (CIP) at 37 °C for 1 h to prevent vector self-ligation.
  6. Purify the digested insert and vector using 1% agarose gel electrophoresis. Visualize the digested insert and vector bands from the ethidium bromide–stained gel by UV irradiation at 254 nm. Excise the gel bands containing the vector and insert and isolate the respective DNA fragments using a gel extraction kit following manufacturer’s protocol; elute DNAs using nuclease-free water.
  7. Use 100 ng of each digested vector and insert DNAs (~1:7 ratio) to set up a 20 μL ligation reaction as described in Recipe 3. Incubate the reaction mixture at room temperature for 1 h followed by incubation at 4 °C for 5–12 h.
  8. Transform 10 μL of the ligation reaction mixture in *E. coli* DH5α competent cells and plate them in LB agar plate containing kanamycin (0.05 mg/mL) antibiotic. A control ligation reaction and transformation could be performed without the insert DNA in the reaction mixture. The control reaction should result in significantly fewer (~10-fold) colonies than the ligation with the insert.
  9. Isolate plasmid DNA from the colonies obtained upon transformation using a plasmid isolation kit and confirm the positive clones by sequencing.
- **Molecular cloning of type III toxin protein**
  1. Design forward and reverse primers to clone the type III toxin into an *E. coli* expression vector under an inducible promoter with a hexahistidine tag. The vector must be compatible for co-transformation with the pRSFDuet-1 vector with a different antibiotic resistance and a different origin of replication (e.g., pCold II, pRSFDuet-1, pET21a(+), etc.) In our study, we cloned the type III toxin into a pCold II vector under a cold-shock inducible promoter with an N-terminal hexahistidine tag between the restriction enzyme sites NdeI and XbaI.
  2. Obtain the ligated plasmid containing toxin insert in the vector of interest by following cloning steps A2–A7 of antitoxin cloning using the toxin insert and appropriate vector DNA.
  3. Co-transform 10 μL of the ligation mixture along with 100 ng of the antitoxin DNA containing plasmid in *E. coli* DH5α competent cells and plate on an LB agar plate containing both kanamycin (0.05 mg/mL) and ampicillin (0.1 mg/mL) antibiotics.
  4. Isolate the plasmid DNA mixture (toxin and antitoxin) from the colonies obtained and confirm the positive toxin clones by sequencing using primers specific to the toxin cloning site. The obtained plasmid DNA mixture will be used further to transform *E. coli* cells to express and purify the type III TA complex. In our study, the plasmid DNA mixture contains antitoxin and toxin cloned in vectors, as shown in Supplementary Table 1.
    **Figure 1.**
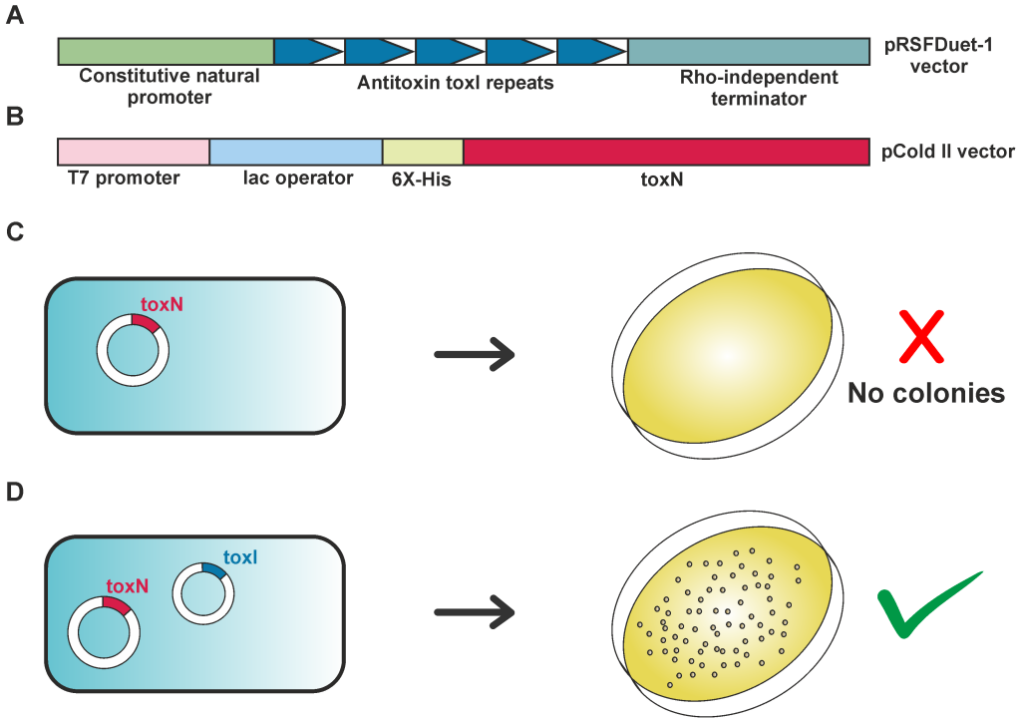
A general strategy for cloning type III toxins in expression vectors. (A) Schematic of cloning of antitoxin ToxI RNA in an expression vector. The antitoxin DNA was cloned along with its natural constitutive promoter in pRSFDuet-1 vector. (B) Schematic of cloning of toxin ToxN protein in an expression vector. The toxin DNA was cloned with an N-terminal hexahistidine tag in an IPTG inducible vector such as pCold II. (C) No colonies were obtained upon transformation of plasmids containing only toxin. (D) Positive colonies were obtained upon co-transforming plasmids containing toxin and antitoxin in different compatible vectors.
- **Transformation and inoculation**
  1. Place a vial of *E. coli* BL21 DE3 competent cells from -80 °C stock on ice to thaw for 15 min.
  2. Add ~100 ng of the plasmid mixture (toxin and antitoxin) and incubate on ice for 20 min.
  3. Provide heat shock by placing the vial in a dry bath at 42 °C for 45 s and immediately transfer the vial on ice and incubate for 5 min.
  4. Add 250 μL of LB medium to the vial in a laminar airflow cabinet and incubate for 1 h in a shaker incubator maintained at 37 °C at 180 rpm.
  5. Spread plate by using 150 μL of this culture on an agar plate containing double antibiotic [corresponding to the plasmid combination chosen; here, we used ampicillin (0.1 mg/mL) and kanamycin (0.05 mg/mL)] and incubate for 12–14 h to obtain the transformed colonies. We did not observe bacterial colonies post-transformation of only toxin plasmid (Figure 1C). However, the co-transformation of toxin and antitoxin plasmids resulted in bacterial colonies selected using dual antibiotics (Figure 1D).
  6. Inoculate 100 mL of LB medium with both antibiotics with a single colony from the transformed plate. Incubate overnight at 37 °C at 180 rpm in a shaker incubator.
  7. Depending on the plasmid in which the toxin and antitoxin are cloned (Table 1), one of the following steps can be followed:
    - Toxin cloned in pCold II: inoculate 10 mL of the overnight primary culture (in LB media) into a larger secondary culture (1 L of LB media) and incubate until OD_600_ reaches ~0.8–1.0. Incubate the culture at 15 °C without shaking for 30 min and induce by adding IPTG to a final concentration of 1 mM. Incubate at 180 rpm for 24 h at 15 °C for complex expression. Harvest the cells by centrifugation at 6,800× *g* for 15 min.
    - Toxin cloned in pET-21a(+): inoculate 10 mL of the overnight primary culture (in LB media) into a larger secondary culture (1 L of LB media) and incubate until an OD_600_ ~0.6–0.8. Induce by adding IPTG to a final concentration of 1 mM. Incubate the culture at 180 rpm for 4–5 h at 37 °C for complex expression. Harvest the cells by centrifugation at 6,800× *g* for 15 min.
      **Stop point:** The cell pellet can be stored at -20 °C for up to a month before processing.
- **Cell lysis and Ni-NTA affinity chromatography**
  1. Resuspend the cells in lysis buffer (see Recipes) and add a tablet of protease inhibitor cocktail.
  2. Lyse the cells by sonication with a pulse of 3 s on and 6 s off and an amplitude of 32%.
  3. Centrifuge the lysed cells at 18,328× *g* for 45 min at 4 °C in the Oakridge centrifuge tubes.
  4. Equilibrate Ni^2+^-NTA column connected to a peristaltic pump with lysis buffer. Filter the supernatant using a 0.45 μm syringe filter and load it onto the column at a 1.5 mL/min flow rate. After loading, wash the column with 50 mL of wash buffer (see Recipes). Elute the complex using elution buffer.
  5. Collect three fractions: E1, the first 2 mL; E2, the next 15 mL; and E3, the final 5 mL. Store at 4 °C.
  6. Wash the column with the final wash buffer to remove any bound protein to the column. Pass 25 mL of Milli-Q water and store the Ni^2+^-NTA column in 20% ethanol.
  7. PAGE analysis of toxin and antitoxin: to analyse the flowthrough, wash, and elution fractions on 12% SDS-PAGE for the presence of toxin protein and on 15% Urea-PAGE for the presence of RNA antitoxin. Visualize the protein in SDS-PAGE gels with Coomassie brilliant blue staining solution followed by destaining using destaining solution (see Recipes). The RNA can be visualized by staining the urea-PAGE gels with 0.25% toluidine blue solution followed by destaining using water.
- **Dialysis**
  1. Collect all the elution fractions that contain the TA components for dialysis (usually, E2 has the TA components).
  2. Dialysis: wash an appropriate length (which can hold the elution fraction volume containing the TA component) of the 6 M dialysis bag with a cutoff of 3.5 kDa with MilliQ H_2_O and equilibrate with dialysis buffer.
  3. Carefully add the elution fractions into the bag and dialyse at 4 °C for 4–5 h.
  4. Change the dialysis buffer and continue to dialyse for 12–14 h.
- **Ion-exchange chromatography**
  1. Equilibrate the anion-exchange column with 25 mL of ion-exchange buffer A.
  2. Filter the dialysed complex using a 0.45 μm syringe filter and load it onto the anion-exchange column at a 1 mL/min flow rate connected to ÄKTA prime plus FPLC system. After loading, wash the column with 20 mL of ion-exchange buffer A.
  3. Elute the complex by increasing the gradient of NaCl from 50 to 1,000 mM (using buffer A and buffer B), over a volume of 100 mL. This should yield separate fractions of ToxN protein (~0%–20% buffer B), TA RNP complex (~45%–55% buffer B), and antitoxin ToxI RNA repeat (~55%–75% buffer B) (Figure 2A), which can be confirmed by PAGE analysis of protein and RNA components.
    **Figure 2.**
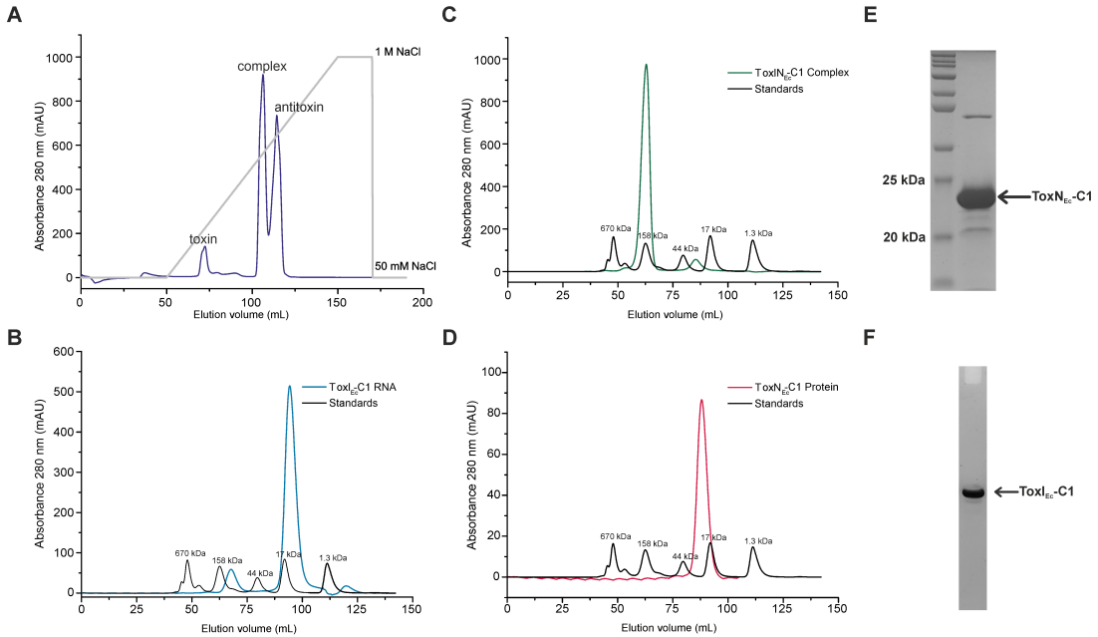
Expression and purification of type III toxin–antitoxin (TA) components from ToxIN_Ec_-C1. (A) Anion-exchange chromatography profile of ToxIN_Ec_-C1 complex shows the purification of individual toxin, antitoxin, and complex components of ToxIN_Ec_-C1. (B–D) Size exclusion chromatography (SEC) profiles of ToxI_Ec_-C1 RNA (B), ToxN_Ec_-C1 protein (C), and ToxIN_Ec_-C1 complex (D). The SEC profiles suggest the presence of the purified components in a single oligomeric state. (E) SDS-PAGE analysis of SEC-purified ToxIN_Ec_-C1 complex. Gel is stained using Coomassie stain for protein and shows the presence of ToxN_Ec_-C1 protein. (F) Urea-PAGE analysis of SEC-purified ToxIN_Ec_-C1 complex. Gel stained using toluidine blue dye for RNA shows the presence of ToxI_Ec_-C1 RNA repeat.
- **Size exclusion chromatography (SEC)**
  1. After ion-exchange chromatography, collect the fractions containing toxin, antitoxin, and complex separately.
  2. Pool each set of fractions separately and concentrate using Amicon^®^ Ultra-15 centrifugal filter (3.5 kDa) to a final volume of ~4 mL.
  3. Filter the concentrated samples using a 0.45 μm syringe filter and inject into Sephacryl S-200 or Superdex S200 columns, pre-equilibrated with SEC buffer. Figure 2B–2D show the SEC elution profiles of antitoxin RNA (Figure 2B), TA RNP complex (Figure 2C), and toxin protein (Figure 2D).
  4. Analyse the fractions on SDS-PAGE and urea-PAGE. Figure 2E and 2F show the SDS-PAGE and urea-PAGE analysis of SEC-purified ToxIN_Ec_-C1 complex for the presence of toxin protein and antitoxin RNA, respectively.
    The fractions can be pooled together and concentrated appropriately depending on the experiment that needs to be performed, such as NMR spectroscopy, crystallization, or ITC. Using this protocol, we could also successfully purify two other ToxIN complexes and their components (from cluster 4 and 5 of *E. coli*) (Manikandan et al., 2022) (Supplementary Figure 1 and Supplementary Figure 2).

## General notes and troubleshooting

1. The complete type III TA operon should contain i) the predicted natural promoter (including the -35 and -10 box regions), ii) the antitoxin RNA repeats, iii) the predicted transcription terminator, and iv) the toxin protein coding region (codon optimized for *E. coli* expression). In our study, the type III TA operon from *E. coli* ToxIN Cluster 1 (ToxIN_Ec_-C1) including natural promoter was synthesized and cloned into a pUC57 vector by GenScript (USA).
2. It is advisable to run the fractions obtained after every step of purification (Ni^2+-^NTA affinity chromatography, ion-exchange chromatography, and size exclusion chromatography) on the SDS-PAGE and Urea-PAGE to visualize the protein and RNA, respectively. This also ensures the visualization of impurities in the fractions eluted and whether another round of purification is required before proceeding to the biophysical experiments.
3. All the buffers used in the protocol were filtered with 0.22 μm sterile filters and used within one week of preparation.
4. For measuring the concentration of protein, any of the standard methods could be employed, such as absorbance at 280 nm or Bradford’s assay.
5. Absorbance at 260 nm was used for measuring the concentration of RNA.
6. In cases where it was difficult to measure the protein concentration accurately, 1:1 ratio of the RNA:protein was assumed to obtain an approximate concentration of the RNP TA complex.
7. Since the protocol involves the purification of RNA and RNA–protein complexes, it is important to be careful while handling the samples and to keep the columns away from RNases to obtain a good yield.
8. The toxin and antitoxin of ToxIN systems used in our study were cloned in separate compatible vectors. It is also possible to clone them in the same vector under different promoters to express and purify the components.
9. The toxin protein and TA complex fractions can be stored at 4 °C (up to a week) and the antitoxin RNA fractions can be stored at -20 °C (up to a month) or -80 °C (for more than a month).

## Supplementary information

The following supporting information can be downloaded here:

1. Supplementary Figure 1. Expression and purification of type III TA components from ToxINEc-Cluster 4.
2. Supplementary Figure 2. Expression and purification of type III TA components from ToxINEc-Cluster 5.
3. Supplementary Table 1. Table showing the details of source E. coli strains of the ToxIN systems studied and plasmid vectors used to express and purify different ToxIN complexes.
